# Exploration of Genome-Wide Recombination Rate Variation Patterns at Different Scales in Pigs

**DOI:** 10.3390/ani14091345

**Published:** 2024-04-29

**Authors:** Zuoquan Chen, Meng Zhou, Yingchun Sun, Xi Tang, Zhiyan Zhang, Lusheng Huang

**Affiliations:** National Key Laboratory for Swine Genetic Improvement and Germplasm Innovation, Jiangxi Agricultural University, Nanchang 330045, China

**Keywords:** recombination, pigs, hotspots, *PRDM9*, population genetics

## Abstract

**Simple Summary:**

The study constructed and compared the broad-scale meiotic recombination maps using populations from four different breeds of Western and Chinese pigs to investigate similarities and differences. At the broad scale, we found that the recombination patterns among different pig breeds are similar, with variations in recombination intensity and locations. Particularly, the differences in recombination patterns between Western and Chinese pigs are more significant compared to within-group variations. Furthermore, this study utilized 16 million SNP variants from unrelated individuals for the first time to construct a fine-scale historical recombination map. At the fine scale, we identified potential recombination hotspots and coldspots and verified the known features of these recombination hotspots and coldspots. Subsequently, by analyzing the overlap between recombination hotspots and regions of TSSs and H3K4me3 peaks, we found that recombination hotspots in pigs are distanced from gene TSSs and potential active promoters. Therefore, we propose for the first time evidence suggesting that recombination hotspots in pigs are regulated by *PRDM9*, providing a meaningful contribution to enhancing the efficiency of genomic selection breeding and further understanding the molecular mechanism of recombination in pigs.

**Abstract:**

Meiotic recombination is a prevalent process in eukaryotic sexual reproduction organisms that plays key roles in genetic diversity, breed selection, and species evolution. However, the recombination events differ across breeds and even within breeds. In this study, we initially computed large-scale population recombination rates of both sexes using approximately 52 K SNP genotypes in a total of 3279 pigs from four different Chinese and Western breeds. We then constructed a high-resolution historical recombination map using approximately 16 million SNPs from a sample of unrelated individuals. Comparative analysis of porcine recombination events from different breeds and at different resolutions revealed the following observations: Firstly, the 1Mb-scale pig recombination maps of the same sex are moderately conserved among different breeds, with the similarity of recombination events between Western pigs and Chinese indigenous pigs being lower than within their respective groups. Secondly, we identified 3861 recombination hotspots in the genome and observed medium- to high-level correlation between historical recombination rates (0.542~0.683) and estimates of meiotic recombination rates. Third, we observed that recombination hotspots are significantly far from the transcription start sites of pig genes, and the silico–predicted *PRDM9* zinc finger domain DNA recognition motif is significantly enriched in the regions of recombination hotspots compared to recombination coldspots, highlighting the potential role of *PRDM9* in regulating recombination hotspots in pigs. Our study analyzed the variation patterns of the pig recombination map at broad and fine scales, providing a valuable reference for genomic selection breeding and laying a crucial foundation for further understanding the molecular mechanisms of pig genome recombination.

## 1. Introduction

Meiotic recombination is the exchange of genetic materials that occurs between homologous chromosomes during meiosis. It plays crucial roles in genetic variation, haplotype structure, and the generation of novel phenotypes in eukaryotic organisms undergoing sexual reproduction. However, the recombination rate varies significantly across species, populations, genders, and individuals [1]. Understanding how recombination rates vary subtly between various species, populations, and even individuals is of significant importance to elucidating the molecular evolution of genomes influenced by this process [2].

The continuous advancement of sequencing technology has led to an increasing number of researchers utilizing whole-genome sequencing data and/or population-based recombination estimation methods to construct precise recombination landscapes across various species [3,4,5]. Studies have shown that there are extensive common features in the genome-wide recombination landscape of vertebrates, such as higher recombination rates in females than in males, shorter chromosomes having higher average recombination rates [6,7], and significant correlations existing between recombination rates and DNA features such as GC content, specific motifs, and transcription start sites (TSSs) [8]. Pigs are considered one of the most prolific meat-producing livestock globally. Archibald et al. [9] constructed the first genome-wide linkage map of pigs in 1995, subsequently utilizing an increasing number of mutations to develop denser linkage maps. However, current research predominantly relies on 50–80 K chip data [7]. The variation and influencing factors of recombination across Western pig breeds (Duroc, Landrace, Large White, Pietrain, and hybrids) were investigated by Cathrine Brekke et al. using medium- and high-density microarray data [10]. They found that recombination rates in pigs differ between breeds, sexes, and individuals and that individual crossover counts are associated with the *RNF212*, *SYCP2*, and *MSH4* genes in pigs.

Recombination in numerous organisms is concentrated within specific and localized regions commonly referred to as “hotspots” [11]. Recombination rates at large scales (Mb) tend to be conserved over longer evolutionary timescales, but recombination rates at the fine scale (Kb) within chromosomes can evolve rapidly [5]. Local variations in recombination within a population, particularly the spatial distribution of hotspots, can exert significant impacts on population evolution and genetic diversity. Recombination hotspots are thought to be inherently unstable because of a “hotspot drive” mechanism that continuously replaces alleles favoring higher recombination rates [12]. The recombination “coldspots” are usually located near the centromere of the chromosome, and the recombination rate is relatively low. In these regions, genotype and genome structure are relatively stable. Recombination coldspots play a crucial role in preserving the stability of genome structure, mitigating the occurrence of deleterious recombination events and upholding the genetic equilibrium within populations [13]. The comprehensive understanding of the intricate recombination landscape within recombination hotspots is crucial for deciphering the patterns of recombination rates and unraveling the underlying mechanisms governing recombination regulation.

Previous studies have suggested that the position of hotspots in the genome and their mutation rates depend on the presence of an active *PRDM9* gene within the species’ genome [14]. When present and possessing intact functional domains (for example, in humans [15] and mice [16]), *PRDM9* coordinates the recombination landscape by binding its rapidly evolving zinc finger array to specific nucleotide motifs, leading to changes in the H3K4me3 mark and coordinating recombination away from genes and their functional regions [17]. On the contrary, vertebrates lacking functional *PRDM9* genes (such as birds [18] and canids [19]) exhibit recombination hotspots concentrated in promoter regions. As a mammal, the genome of pigs contains the full-length *PRDM9* gene. To our knowledge, there is currently no research indicating whether the recombination hotspot pattern in pigs is regulated by *PRDM9*. Constructing a fine-scale recombination map of pigs is of significant importance for exploring the regulatory patterns of recombination hotspots in pigs.

The primary objectives of this study were to conduct a comprehensive comparison of the large-scale recombination map among various pig breeds and analyze the genomic characteristics of the fine-scale recombination landscape to reveal potential mechanisms. We constructed a recombination map using large-scale pedigree and middle-density genotype data from four different pig breeds, and then conducted a comparative analysis to identify the similarities and differences among these breed-specific recombination landscapes. We employed a population-based approach utilizing whole-genome sequencing data from 36 unrelated individuals to construct fine-scale recombination landscapes and identify possible intervals of recombination hotspots and coldspots, which helped to address several outstanding questions.

## 2. Materials and Methods

### 2.1. Sample and Genotyping

The samples used in the pedigree-based recombination landscape investigation were four populations produced in our laboratory previously: the Sutai population in Jiangsu, China [20]; the Two End Black (Two_black) population in Jiangxi, China [21]; the Erhualian and white Duroc hybrid population (EH × WD) [22]; and a three-generation hybrid population produced by crossing Duroc, Landrace, and Yorkshire (D × L × Y) [23]. For these animals, we only selected focal individuals (FIDs) with at least one known parent and at least two offspring, as well as individuals with unknown parents but at least four offspring, for our analysis [24]. According to the classification criteria of FIDs, the individual numbers and FID numbers each population are summarized in Table 1. A total of 3279 individuals from the four populations were genotyped using the Illumina PorcineSNP60 BeadChip according to the manufacturer’s protocol. The physical locations of SNPs on chromosomes were assembled with reference to the porcine reference genome sequence (Sus_scrofa11.1) (http://asia.ensembl.org/Sus_scrofa/Info/Index accessed on 23 March 2023). Plink V1.9 [25] was used to perform quality control to exclude individuals with call rates below 95% and SNPs with call frequencies < 98%, and sex chromosome information was not used for recombination detection.

The population-based refined recombination estimation used whole-genome sequence data from 36 unrelated individuals comprising 3 breeds of purebred Duroc, Landrace, and Yorkshire. Briefly, BGI’s MGI sequencing platform was used to build a library for sequencing (150 bp × 150 bp paired-end sequencing) with an average coverage depth of 10×. Variants were subsequently called following the best-practice pipeline of the Genome Analysis Toolkit [26,27] (GATK, https://gatk.broadinstitute.org, v4.1, accessed on 23 March 2023) using pig reference gene sequence assembly (Sus_scrofa11.1). To prevent bias in high-copy-number variants or poorly sequenced regions, we first filtered variants based on total sequencing depth (filter variants that were less than one-third and greater than twice the population average sequencing depth) and performed QC using the “--minQ 30, --max-alleles 2, max-maf < 0.05” parameters of Vcftools [28] to filter low-quality, multi-allelic, and minor-allele-frequency variants of less than 5%; we finally kept 16.5 million SNPs for subsequent analysis.

### 2.2. Pedigree-Based Recombination Rate Estimation

In order to accurately estimate the pedigree-based [6] recombination rates (meiotic recombination rates) among different breeds, we referred to the ideas of Petit et al. [24]. Briefly, we firstly performed quality control to correct potential errors by exhibiting the distribution of recombination crossover events using LINKPHASE3 [29]. Due to the unreliability of double crossovers occurring within less than 3 Mb in the same meiotic event, we set the positions of double crossover recombination and corresponding genotypes of individuals as missing. We then re-used LINKPHASE3 to construct their paternal and maternal phases and to detect recombination crossovers chromosome by chromosome. The meiotic recombination rate within 1 Mb windows was next estimated using the Poisson distribution and Monte Carlo sampling strategy [24]. Additionally, we obtained the average meiotic recombination rate data for males and females estimated by previous researchers using nine different breeds [7].

### 2.3. Population-Based Recombination Rate Estimation

To estimate population-based [6] recombination rates (historical recombination rates), we first used smc++ [30] and a porcine reference gene sequence assembly (Sus_scrofa11.1) to infer demographic histories with a mutation rate per base per generation of 1.2 × 10^−9^ following previous studies [31]. We then estimated the historical recombination rate using the default parameters of pyrho [32]. Pyrho uses SNPs as intervals with a step size of one SNP. Furthermore, to evaluate the recombination rate with different window sizes and methods, we also estimated historical recombination rates using a 10 kb window and a 5 kb step size for FastEPRR [33]. Following the software authors’ recommended parameters, windows were excluded if they overlapped with known sequencing gaps in the pig reference genome (Sus_scrofa11.1) or if the number of non-singleton polymorphic sites was less than 10. To more precisely estimate the recombination rate, we converted the population parameters to ms [34] per the software matching values of smc++ to estimate the population history.

### 2.4. Identification of Recombinant Coldspots and Hotspots and Their Genomic Characterization

We used a “filtering” approach to identify coldspots and hotspots from recombination rate results estimated by pyrho according to the method described by Wooldridge et al. [5]. In brief, we considered consecutive SNPs with a recombination rate greater than 10 times the mean chromosomal recombination rate as potential recombination hotspots. Recombination intervals with less than two SNPs and interval lengths greater than 5 kb were then filtered to reduce false positives. Hotspots separated by 2 SNPs with an interval ≤1 kb were merged together. Similarly, we took the intervals that were less than 1/10 of the average chromosome recombination rate and contained at least 3 SNPs as candidate coldspots.

To analyze the differences in the distribution characteristics of recombination coldspots and hotspots, we calculated the nucleic acid diversity of the population and the nucleic acid diversity of recombination coldspot and hotspot intervals using VCFtools [28] and BEDtools [35], respectively. Also, to see the GC bias of recombinant coldspots and hotspots, we extracted coldspot and hotspot interval sequences from the reference genome separately using SeqKit [36] and the stats for their GC contents. We generated 1000 sets of randomly selected genomic fragments matching the size of recombination coldspots and hotspots as controls to test their significance.

### 2.5. Characterizing the Genomic Distribution of Hotspots and Functional Enrichment Analysis

The BEDtools [35] “intersect” function was used to discover hotspots that overlapped with 3 kb anterior and posterior to the TSS of annotated genes. A hotspot was considered to overlap with a TSS when the midpoint of the hotspot overlapped with any part of the 6 kb TSS region. Only when the number of position overlaps of recombination hotspots was greater than that of the random point dataset did we consider the recombination hotspots to be significantly enriched in the TSS region. Similarly, we evaluated the number of random points overlapping with gene TSS locations in a dataset of 200 random points to test the enrichment significance. To further analyze Gene Ontology (GO) terms and KEGG pathways associated with genes hosting promoters that are approached by recombination hotspots, the above genes were analyzed for functional enrichment using the g:Profile server [37].

### 2.6. Analysis of H3K4Me3 Chip-Seq Data

Previous research has confirmed that trimethylated histone H3 lysine 4 (H3K4me3) is usually found around the TSSs of genes and targets active promoters [38]. To further analyze the overlap between recombination hotspots and H3K4me3 peaks positions, we downloaded the H3K4me3 chip-seq data of pig kidney cells from the CNCB database (ID: CRA006465 [39]) with the aim of identifying potential active promoters in the pig genome. We conducted the chip-seq data analysis following these steps: (1) trimming and filtering of reads using fastp [40]; (2) mapping chip-seq data to the pig genome (Sscrofa11.1) in paired-end mode using hisat2 v2.2.0 [41]; (3) processing, sorting, and filtering alignment files using SAMtools [42]; (4) removing PCR duplicates using picard (http://broadinstitute.github.io/picard/, accessed on 23 March 2023); and (5) identifying H3K4me3 peaks using MACS v2.1.0 [43].

### 2.7. Motif Analysis

We used the online tool ExPASy [44] to translate the DNA sequence of the *PRDM9* gene in the reference genome into a protein sequence, and then used it as the input of the Cys 2 His 2 zinc finger prediction tool (http://zf.princeton.edu/index.php, accessed on 23 March 2023) [45]. This software de novo predicted the DNA binding specific position weight matrix (PWM) of the Cys 2 His 2 zinc finger array. The domain was selected to predict the DNA binding motif when the HMMER bit score of the zinc finger domain was not less than 17.7. Finally, we used AME 5.5.2 [46] to determine whether the *PRDM9* zinc finger DNA recognition motif was enriched in hotspot sequences relative to the recombinant coldspot sequence computer prediction. Furthermore, to analyze recombinant hotspot DNA sequences for the presence of enriched DNA motifs and motif characterization, we identified DNA motifs enriched in the hotspot intervals by scanning the hotspot sequences by chromosome using MEME 5.5.2 [47], which recognizes motifs in the range of 6–50 bases by default and terminates after identifying up to 50 likely motifs to minimize duplicate identifications.

## 3. Results

### 3.1. Comparison of Large-Scale Meiotic Recombination Maps among Different Pig Breeds

**Figure 1:**
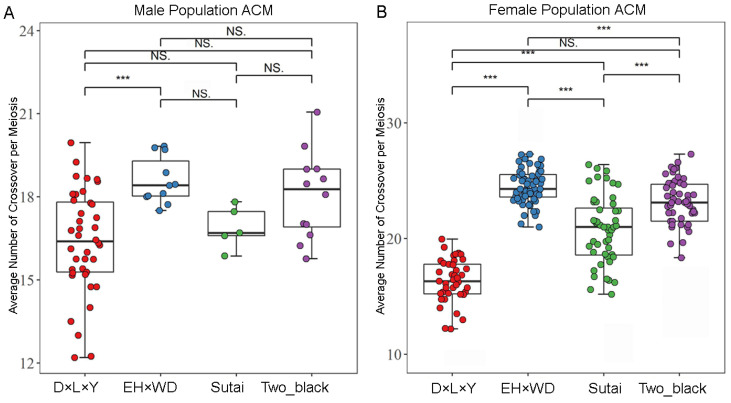
Comparison of scatter plots of the ACM among different populations by gender. (**A**) Scatter-overlay boxplots of the average number of meiotic crossover events per division among different populations of males. The X-axis represents different populations; the Y-axis represents the ACM. “*” indicates a *p*-value < 0.05; “**” indicates a *p*-value < 0.01; “***” indicates a *p*-value < 0.0001; NS indicates no significant difference. (**B**) Scatter-overlay boxplots of the average number of crossovers per meiotic crossover event among different populations of females.

We estimated meiotic recombination in domestic pigs using pedigrees from four breeds. We, in total, detected 38,332 crossovers in 2158 meiosis of 71 paternal FIDs, and 43,061 crossovers in 1865 meiosis of 188 maternal FIDs in the four populations (Table S1, Figure S1). The average genetic map of male and female was 17.763 M (0.78 cM/Mb) and 23.089 M (1.02 cM/Mb), respectively, which is similar to previous studies in Western commercial pigs [7]. The population average recombination rate and patterns of recombination along the chromosomes were similar across breeds of the same sex, but both male- and female-averaged recombination rates and patterns of recombination along the chromosomes differed significantly (Figure S2). By comparing the average number of crossovers per meiosis (ACM) between the two genders separately, significant differences were observed among all female breeds, except between D × L × Y and Two_black (F-test, *p* = 0.514). In males, however, except for significant differences between D × L × Y and EH × WD breeds (F-test, *p* = 0.02), no other differences reached significance (Figure 1A,B). In order to measure the correlation of 1 Mb window recombination maps among different populations, we conducted a correlation analysis. The results show that the correlations ranged from 0.642 to 0.894, where the correlation coefficient between Sutai with D × L × Y was the smallest, and the correlation coefficient between EH×WD with Two_black was the largest (Table 2). To compare the correlation of Chinese and Western domestic pigs, we grouped them by breed and compared the within-group and between-group correlations. The results show that the correlation between Chinese and Western domestic pigs was lower than the within-group correlation (*t*-test, *p* < 0.05), suggesting greater disparities in recombination patterns between Chinese and Western pigs.

### 3.2. Fine-Scale Recombination Landscapes Using Whole-Genome Sequencing Data

**Figure 2:**
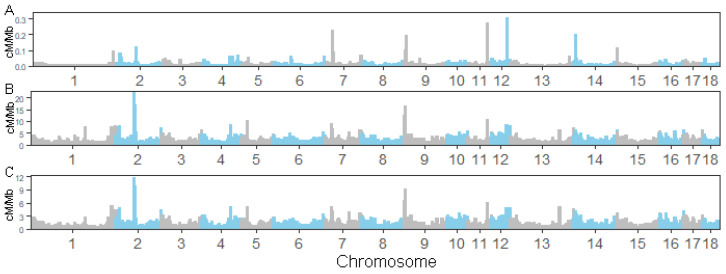
Autosomal recombination landscapes constructed by different software and parameters. From (**A**–**C**), the 1 Mb window recombination rates of pryho, FastEPRR 5 kb window, and FastEPRR 10 KB window, respectively.

First, we obtained the population historical statistics of 36 unrelated individuals (Figure S3). Then, we constructed a historical recombination map based on the LD pattern of unrelated individuals with a resolution of approximately 137.7 bp (Figure S4). In addition, we constructed historical recombination maps at FastEPRR 5 Kb and 10 Kb resolutions (Figures S5 and S6). The results show that at a fine scale, different software and parameters exhibited similar recombination characteristics within the same position interval, but the estimated value of the recombination rate decreased as the resolution increased. At the 1 MB scale, we further compared the estimated recombination rates of different software and parameters. The results show that the parameters had little impact on the estimation of the recombination rate (Spearman rank correlation between FastEPRR5K and FastEPRR10K, rho = 0.975, *p* < 0.001). (Figure 2). Additionally, there was a high correlation in the estimation of the 1 MB recombination rate among different software tools (correlation between FastEPRR 5K, FastEPRR 10K, and pyrho recombination rate: rho = 0.775, *p* < 0.001 and rho = 0.834, *p* < 0.001), which implies the robustness and correctness of our results. Similarly, we also compared the correlation between historical recombination rates and meiotic recombination. However, the results revealed significantly different correlation levels (*p* = 4.9 × 10^−5^, *t*-test) between them across different software platforms (pyrho: rho ∈ 0.542~0.683, FastEPRR: rho ∈ 0.277~0.460) (Table 3). This reflects the similarity in estimating recombination rates between population-based and pedigree-based approaches.

### 3.3. Genomic Characterization of Recombination Coldspots and Hotspots

**Figure 3:**
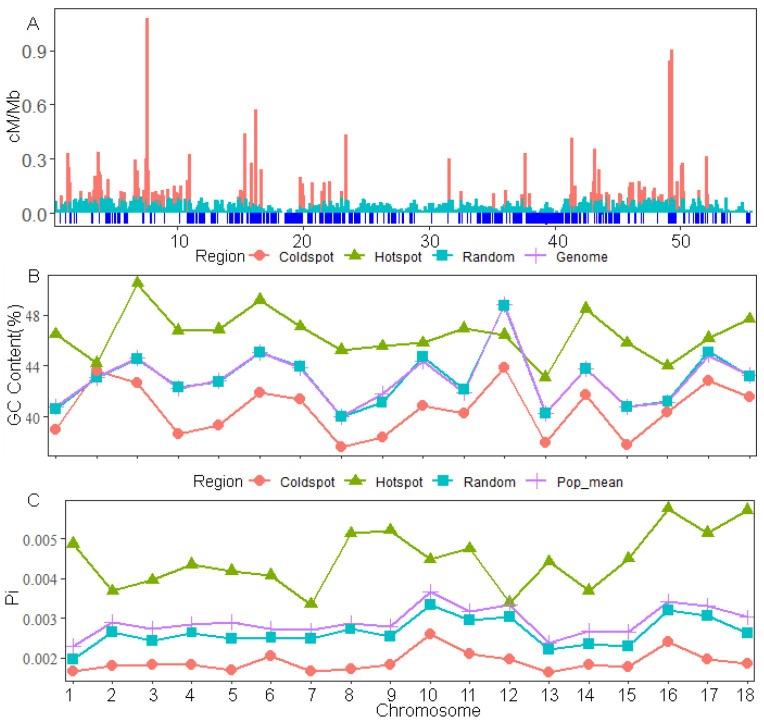
Chromosome distribution of recombination coldspots and hotspots. Comparison of GC content and Pi in coldspot and hotspot intervals. (**A**) Fine recombination map of chromosome 18. The red bar line represents the recombination rate in the possible recombination hotspots, the cyan bar line represents the recombination rate in the non-recombination hotspots, and the blue line below the X-axis represents the location of the possible recombination coldspots. (**B**) Line plots of GC content in different intervals. (**C**) Line plots of Pi in different intervals.

Based on the recombination rates estimated using pyrho, we identified a total of 3861 recombination hotspots and 20,899 recombination coldspots, with an average size of 1.26 kb for the hotspots. The results showed that recombination hotspots were not evenly distributed throughout the genome (Figure 3A and Figure S4), similar to studies in mice and human [48,49]. We also validated the previous hypothesis that, for a given genome size, species with more fragmented karyotypes and more small chromosomes will exhibit higher overall recombination rates and higher GC content [50]. The results showed a significant difference (Wilcoxon test, *p* = 7.63 × 10^−6^) in GC between hotspots and coldspots, with an average of 46.46% GC content in hotspots and 40.53% GC content in coldspots, respectively (average genome-wide GC content level was 42.96%). Comparing to different chromosomes, the GC content in recombination hotspots was higher than that in recombination coldspots in the majority of chromosomes except for chromosomes 2 and 12, and the GC content and the control interval of random spots was between the results of coldspots and hotspots (Figure 3B). It is now generally accepted that the spread of beneficial mutations and the loss of deleterious mutations suppress polymorphism levels due to the existence of a “genetic hitchhiker”, especially in regions of low recombination [51,52]. Similarly, we also validated the previous observations in the comparative analysis of nucleotide diversity within the intervals of recombination hotspots and coldspots. The nucleotide diversity (0.00198 ± 0.0003) in the recombination coldspot region was significantly lower than the nucleotide diversity (0.00291 ± 0.0004) of the recombination hotspot and the population average level (Wilcoxon test, *p* = 3.063 × 10^−8^; Wilcoxon test, *p* = 2.204 × 10^−10^), which was consistent across all chromosomes (Figure 3C). In conclusion, our results demonstrate our reliable identification of potential recombination hotspots and coldspots in pigs.

### 3.4. Pig Recombination Hotspots Are Distant from the TSS

**Figure 4:**
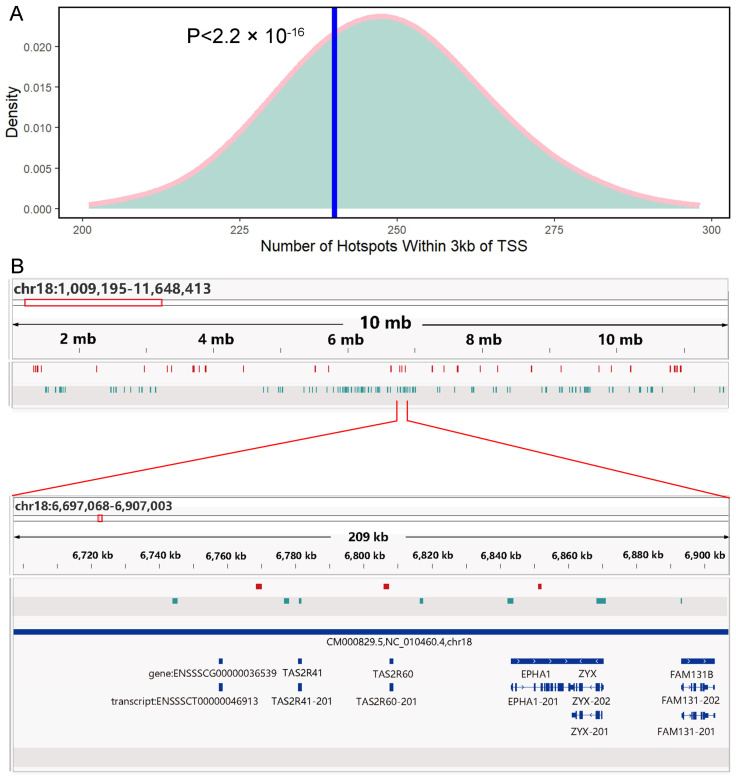
(**A**) Comparison of the number of recombination hotspots and 1000 random points that have intersections near the TSS. The pink line represents the distribution density of the number of random points with intersections near the TSS 1000 times, and the blue line represents the number of recombination hotspots with intersections with the TSS. The *p*-value is calculated by *t*-test. (**B**) Chromosome 18 recombination hotspots and the distribution of H3K4me3 peaks visualized in IGV, with partial regions magnified. Among them, red represents the position interval of the recombination hotspot, green represents the position interval of the H3K4me3 peak, and blue represents the annotation gene of the pig reference genome.

Previous studies have shown that *PRDM9* regulates the recombination landscape by binding to specific nucleotide motifs, which led to changes in the states of H3K4me3 and recombination distant from genes and functional regions [16,17]. In contrast, vertebrates lacking or having only non-functional *PRDM9* (e.g., birds [53] and canines [19]) have recombination hotspots concentrated in promoter regions, possibly because SPO11 defaults to an open region near gene promoters. To explore whether the recombination hotspots in pigs are enriched in TSS regions, we analyzed overlaps of recombination hotspots and TSSs. We found that 240 (6.2%) out of 3861 recombination hotspots overlapped with the 3 Kb region of the TSS, which was significantly lower than the results of 1000 simulated “random points” (Figure 4A). This suggests that *PRDM9* may play a role in determining pig recombination hotspots. Next, we downloaded H3K4me3 chip-seq data from a public database and directly analyzed the overlap of H3K4me3 broad peaks with recombination hotspot intervals. We identified a total of 17,965 reliable H3K4me3 peaks, with an average peak width of 1648.1 bp, among which 11,404 (63.5%) peaks overlapped with the 6 Kb interval of TSSs based on the annotation file. The results show that recombination hotspots overlapped with H3K4me3 peaks to a lesser extent (Figure S7). Figure 4B illustrates the distribution of recombination hotspots on chromosome 18 in relation to the broad peaks of H3K4me3. We randomly zoomed in to a portion of the figure, and it is evident that there was minimal positional overlap between recombination hotspots and potential active promoters (Figure 4B), further suggesting that pig recombination hotspots are distal from TSSs. In order to further analyze the gene categories and related pathways enriched near the recombination hotspots in pigs, we identified genes that intersected with the recombination hotspots at around 3 Kb of the TSS (240 recombination hotspots intersected with the TSS of 182 genes) to determine enriched GO terms. We identified a total of nine enriched GO terms, primarily related to signal transduction, odor binding, and plasma membrane (Table S2), which is consistent with observations made by previous studies [11].

### 3.5. Motif Analysis

**Figure 5:**
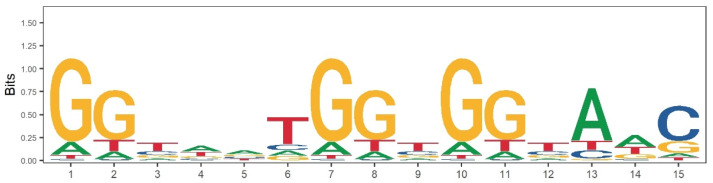
Predicted *PRDM9* gene zinc finger domain 15-mer DNA recognition sequence. Four different colors and letters represent four different bases, respectively.

In order to further evaluate whether the *PRDM9* gene is involved in the formation of recombination hotspots in pigs, we extracted the DNA sequences of the pig reference genome [54] from the identified recombination hotspot and coldspot regions. We compared these regions for the presence of *PRDM9*’s Cys2His2 zinc finger domain DNA recognition motif (Figure 5) and found the relative enrichment of the computer-predicted PRDM9 recognition motif in the hotspot region (*p* = 3.52 × 10^−167^). This is consistent with the results showing recombination hotspots away from TSSs, further confirming the role of the *PRDM9* gene in hotspot determination in pigs. In addition, we identified 17 enriched motifs in the recombination hotspots compared to the recombination coldspot regions (Table S3). The GC content of the top three motifs, with an average GC content of 55.16%, was much higher than the average GC content of the genome (43.3%), which is consistent with the deviation of the GC content of the recombination hotspots.

## 4. Discussion

This study estimated the historical recombination rate of unrelated samples using two mainstream software tools (pyrho and FastEPRR). These two software tools outperform another software, LDhat [55]. However, we found a moderate to high level of correlation between pedigree-based meiotic recombination rates and historical recombination rates estimated by pyrho, whereas the correlation with the rates estimated by FastEPRR was lower. This is because FastEPRR is more conservative in detecting recombination hotspots, and it estimates the average recombination rate of the windows [56]. Combining the characteristics of both software tools, we chose to use pyrho to estimate the recombination rates between SNPs for subsequent identification analysis of recombination hotspots and coldspots. As mentioned by the software authors, FastEPRR may provide unbiased and accurate estimates even with low sequencing coverage or high missing genotype rates. Therefore, in situations of low SNP density, we considered prioritizing FastEPRR.

The content of GC is believed to be associated with an increase in meiotic recombination from yeast to humans [57,58], representing a common pattern. This recombination pattern results in a significantly higher GC content in recombination hotspot compared to coldspot regions. We validated this known observation by comparing the GC content of recombination hotspot and coldspot regions, as well as by conducting enrichment motif analysis in recombination hotspots. However, the molecular mechanisms underlying recombination hotspots and GC content remain insufficiently understood. Previous studies have suggested that high-GC regions are more prone to being affected by enzymes generating cross-recombinogenic DSBs [59]. For example, in certain organisms, multiple mismatch repairs tend to favor the formation of G-C pairs over A-T pairs [60]. Gerton et al. [61] suggest that the positioning of recombination hotspots is governed by certain features in chromosome structure associated with GC enrichment, while the finer location is controlled by transcription factors and chromatin accessibility. The specific molecular mechanisms behind this phenomenon require further and more in-depth research to be elucidated.

Although *PRDM9* has been shown to play a key role in the location and fate of hotspots in various mammalian species such as humans, mice, cattle, sheep, and horses, its expression in canids has been a notable exception because *PRDM9* has been transformed into a pseudogene in these species. Despite the absence of *PRDM9*, the dog recombination map contains recombination hotspots that are highly stable during evolution. This is because canids have *PRDM9* -independent recombination, and their hotspots are found to be enriched in CpG-rich regions upstream of transcription initiation sites, favoring unmethylated CpG islands. There are few reports on whether there is a functional *PRDM9* gene in pigs, and there is no direct evidence that *PRDM9* and pigs are involved in determining pig recombination hotspots. Given that recombination hotspots are significantly distant from the transcription start sites (TSSs) in pigs and the enrichment of *PRDM9* zinc finger domain DNA binding motifs in recombination hotspot sequences, we believe that the results of this study provide compelling evidence that the *PRDM9* gene may also play a role in controlling recombination hotspots in pigs.

## 5. Conclusions

Based on comparisons of large-scale recombination maps among different breeds, we found that the recombination map of pigs is moderately conservative at the 1 Mb scale between different breeds of the same sex, with more pronounced differences in recombination patterns between Western pigs and Chinese domestic pigs. Based on the fine-scale recombination map, we found that the historical recombination rate in pigs is similar to the meiotic recombination rate, and the recombination hotspots in pigs are distant from the TSS region. In summary, our research results elucidate the differences between broad-scale and fine-scale recombination landscapes, emphasizing the important role of the *PRDM9* gene in determining the location of recombination hotspots. This has important implications for further understanding the potential mechanisms underlying recombination rate variation in pigs.

## Figures and Tables

**Table 1:** Pedigree-based recombination rate study sample size.

Population	All Number	P_FID ^1^	P_MNO ^2^	M_FID ^3^	M_MNO ^4^
Sutai	526	5	81.80	52	8.46
Two_black	519	13	32.69	52	7.67
D × L × Y	1020	11	7.67	67	4.08
EH × WD	1214	42	91.00	25	14.67

^1^ Represents the paternal FID; ^2^ represents the mean number of offspring for the paternal FID; ^3^ represents the maternal FID; ^4^ represents the mean number of offspring for the maternal FID.

**Table 2:** Correlation of 1Mb pedigree-based recombination rates.

	Population				
Population	Pre_Results	EH × WD	D × L × Y	Sutai	Two_Black
Pre_results		0.737	0.642	0.695	0.749
EH × WD	0.747		0.725	0.849	0.894
D × L × Y	0.715	0.782		0.716	0.722
Sutai	0.686	0.765	0.664		0.818
Two_Black	0.746	0.809	0.743	0.716	

Note: the upper triangle represents the correlation of recombination rates within 1Mb windows in females, while the lower triangle represents those in males. “Pre_results” represents the estimated recombination rate from the downloaded previous study [7].

**Table 3:** Correlation of population-based and lineage-based recombination rates.

	Population				
Population	Pre_Results	EH × WD	D × L × Y	Sutai	Two_Black
FastEPRR 10 kb	0.460	0.424	0.429	0.357	0.398
FastEPRR 5 kb	0.422	0.340	0.361	0.277	0.321
pyrho SNP	0.683	0.625	0.598	0.542	0.572
Mean	0.522	0.463	0.463	0.392	0.430

Note: “FastEPRR 10 kb” represents the 1 Mb window population recombination rate estimated at a 10 kb window using FastEPRR software. “FastEPRR 5 kb” represents the 1 Mb window population recombination rate estimated at a 5 kb window using FastEPRR 1.0 software. “Pyrho SNP” represents the 1 Mb window population recombination rate estimated at an SNP window using pyrho.

## Data Availability

Data can be requested from the corresponding author via email.

## Supplementary Materials

The following supporting information can be downloaded at: https://www.mdpi.com/article/10.3390/ani14091345/s1, Table S1: Number of meioses, number of crossovers, and length of genetic map for different populations by sex; Table S2: Significantly enriched GO terms and KEGG pathway in hotspots enriched around the TSS; Table S3: Motifs enriched for recombinant hotspot sequences; Figure S1: Differences in mean genetic distances and recombination rates among chromosomes; Figure S2: Whole-genome recombination maps of four different breeds of pigs; Figure S3: Statistical population history of 36 unrelated individuals; Figure S4: The fine recombination map of the SNP window was constructed using pyrho; Figure S5: The fine recombination map of the SNP window was constructed using a FastEPRR 5 Kb window; Figure S6: The fine recombination map of the SNP window was constructed using a FastEPRR 10 Kb window; Figure S7: The number of recombination hotspots overlapping with the broad peaks of H3K4Me3 compared to 1000 randomly simulated points.
