# Supplementary material for: Exploration of Genome-Wide Recombination Rate Variation Patterns at Different Scales in Pigs
Source: Animals (Basel). 2024 Apr 29;14(9):1345. doi: 10.3390/ani14091345 (PMC11083071; doi:10.3390/ani14091345)
Supplement: Supplementary file 1 [file animals-14-01345-s001.zip › animals-2974750-supplementary.pdf]

Exploration of Genome-Wide Recombination Rate Variation Patterns at Different Scales in Pigs

Zuoquan Chen, Meng Zhou, Yingchun Sun, Xi Tang, Zhiyan Zhang * and Lusheng Huang

National Key Laboratory for Swine Genetic Improvement and Germplasm Innovation, Jiangxi Agricultural University, Nanchang 330045, China

Correspondence: bioducklily@hotmail.com

Supplementary Material

Table S1 Number of meioses, number of crossovers, and length of genetic map for different populations by sex.

Population	Sex	Mesis number	CO number	Map Length(M)
EH×WD	Male	1001	18534	18.493
EH×WD	Female	983	24201	23.34
D×L×Y	Male	322	5368	16.38
D×L×Y	Female	102	1742	21.27
Sutai	Male	409	6923	17.05
Sutai	Female	440	9336	20.62
Two_Black	Male	425	7509	17.68
Two_Black	Female	399	9157	22.82
All	Male	2157	38334	17.78
All	Female	2144	48062	22.42

Note: **“**Sutai” means purebred Sutai pig populations; “Two_black” means Two black pig populations; “D×L×Y” means the Duroc, Landrace and Yorkshire hybrid Populations; “EH×WD” denotes the Erhualian and white Duroc hybrid pig populations; “All” represents the sum of all population. Hereafter.

Table S2 Significantly enriched GO terms and KEGG pathway in hotspots enriched around the TSS.

source	term_name	term_id	adjusted_p_value
GO:MF	G protein-coupled receptor activity	GO:0004930	1.19E-35
GO:MF	odorant binding	GO:0005549	0.000148
GO:MF	inhibitory MHC class I receptor activity	GO:0032396	0.015658
GO:MF	trace-amine receptor activity	GO:0001594	0.015658
GO:MF	peptide antigen binding	GO:0042605	0.017215
GO:BP	detection of chemical stimulus involved in sensory perception	GO:0050907	5.39E-32
GO:BP	antigen processing and presentation of peptide antigen	GO:0048002	0.03183
GO:CC	plasma membrane	GO:0005886	3.46E-12
GO:CC	MHC class II protein complex	GO:0042613	0.033771

Table S3 Motifs enriched for recombinant hotspot sequences.

Motif Logo	E-value	Sites	Width
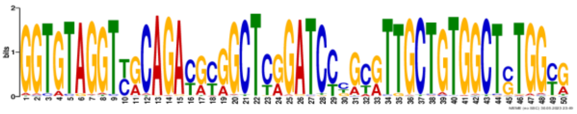	8.6e-111	3923	50
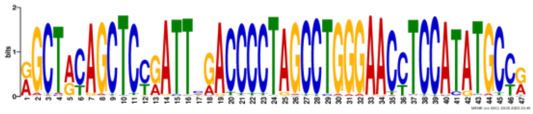	3.4e-120	4564	47
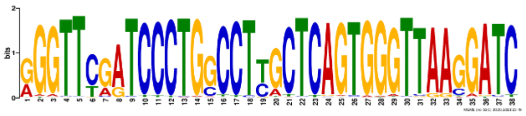	6.1e-103	5192	38
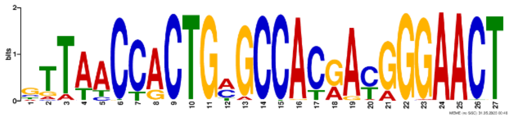	2.7e-073	3817	27
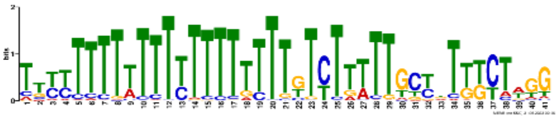	5.4e-077	4005	41
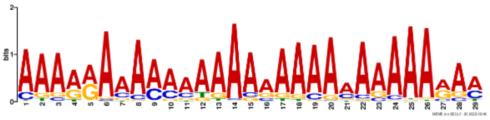	7.2e-052	7067	29
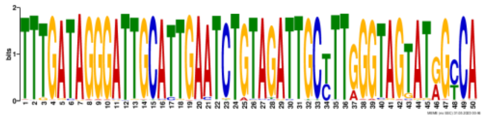	2.0e-049	1085	50
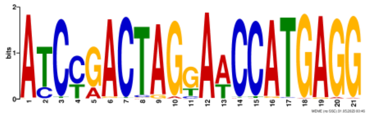	9.4e-036	4548	21
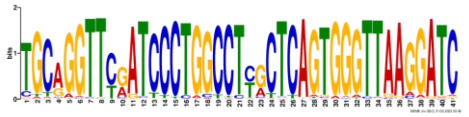	1.1e-055	966	41
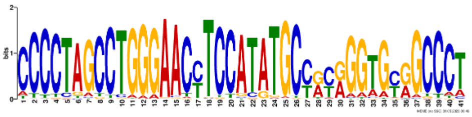	3.2e-041	2735	41
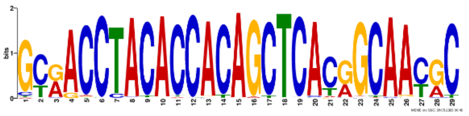	2.5e-034	1710	29
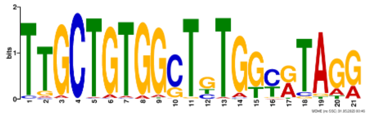	5.9e-024	4317	21
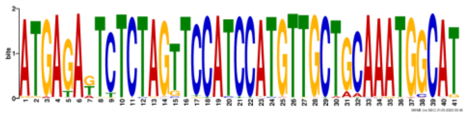	1.2e-023	1086	41
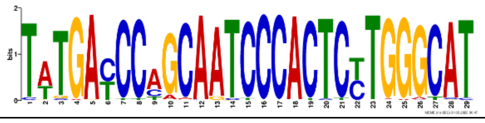	3.3e-022	1599	29

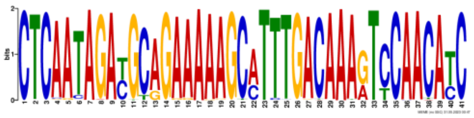	3.3e-035	581	41
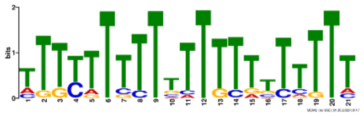	1.2e-021	2717	21
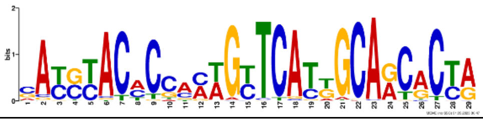	2.0e-021	3725	29

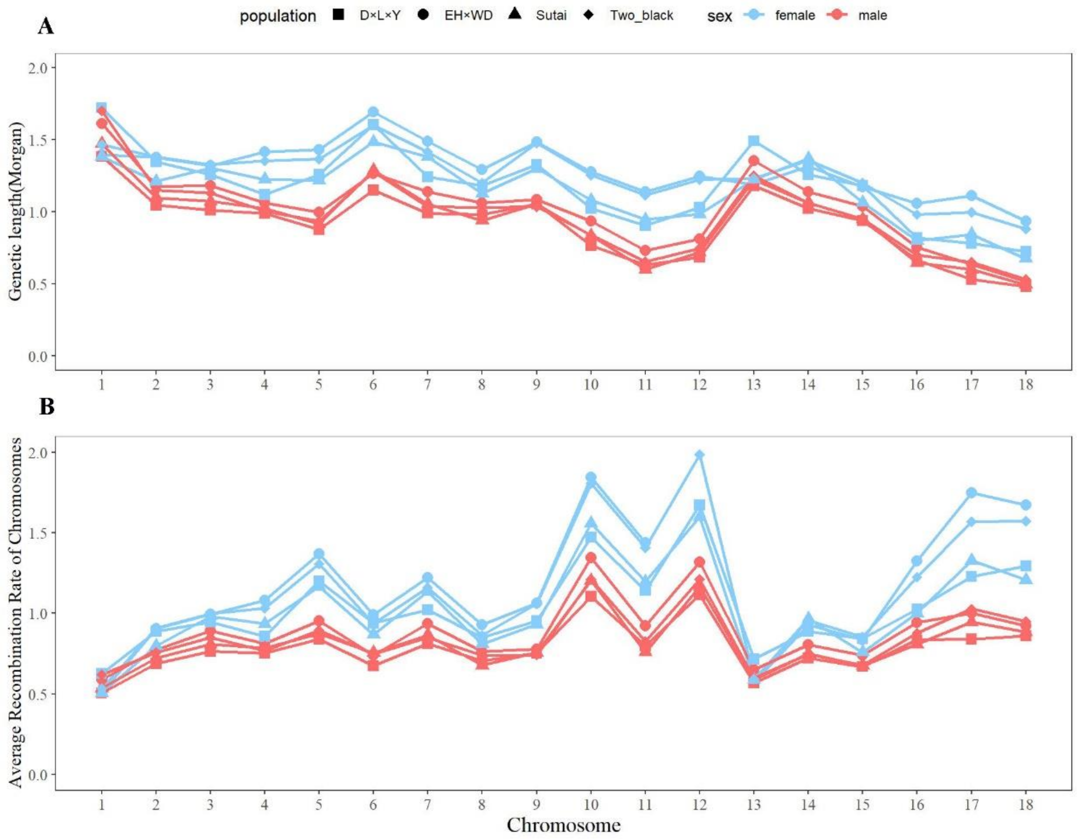

Figure S1 Differences in mean genetic distances and recombination rates among chromosomes. (A) Line plot of the mean genetic length of chromosomes. (B) Line plot of the average recombination rate of chromosomes. Each line represents a pig breed.

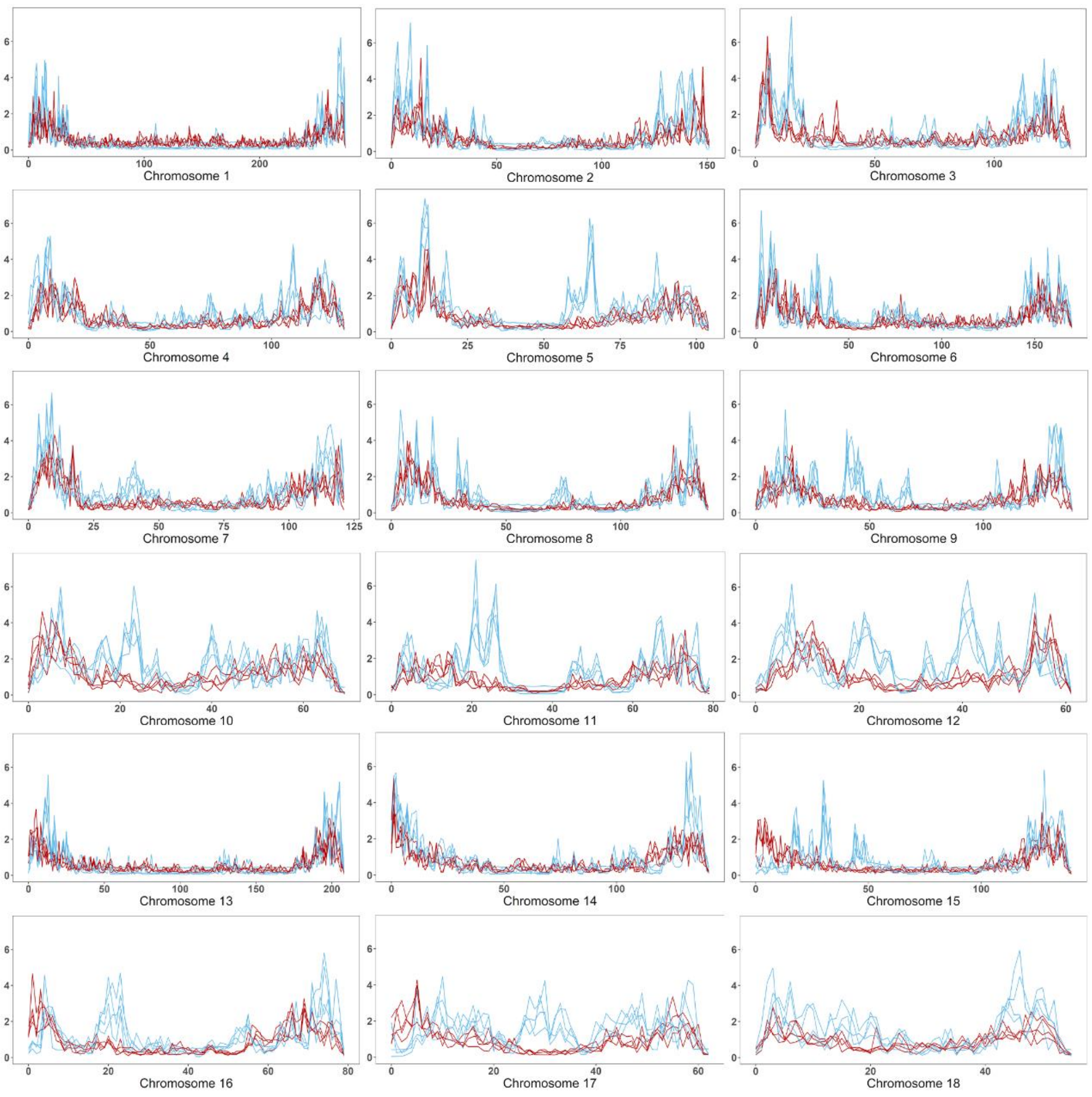

Figure S2 Landscape of recombination in the pig genome. The lines plot shows the recombination rate along a 1 Mb window of the pig genome (Sscrofa11.1). The sky-blue lines are an estimate for females and the red lines are an estimate for males. Each line corresponds to one of the four populations.

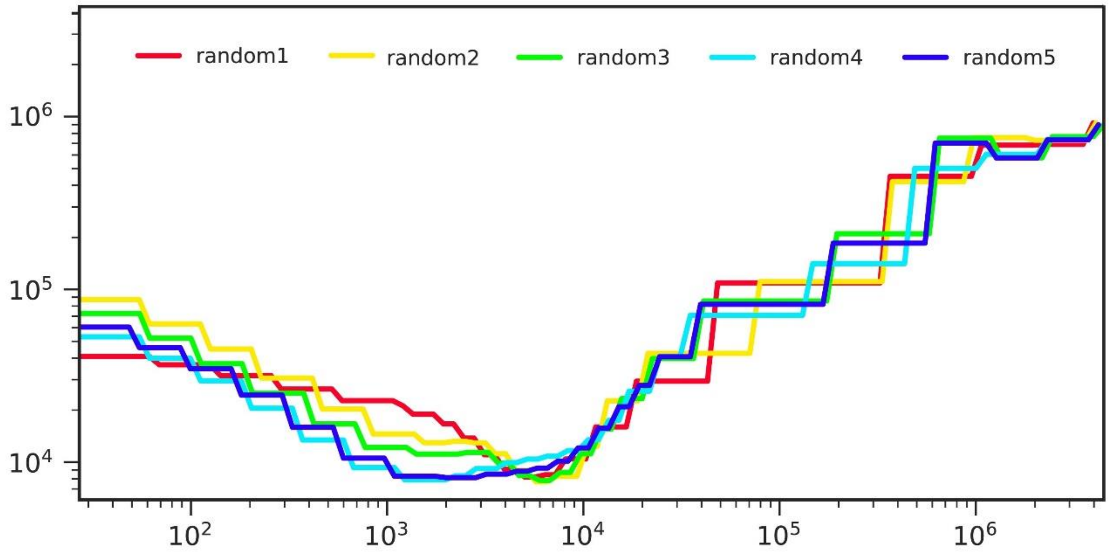

Figure S3 Population statistical history of 36 unrelated individuals. The various colors correspond to distinct and randomly “distinguished” individual estimates of the population history.

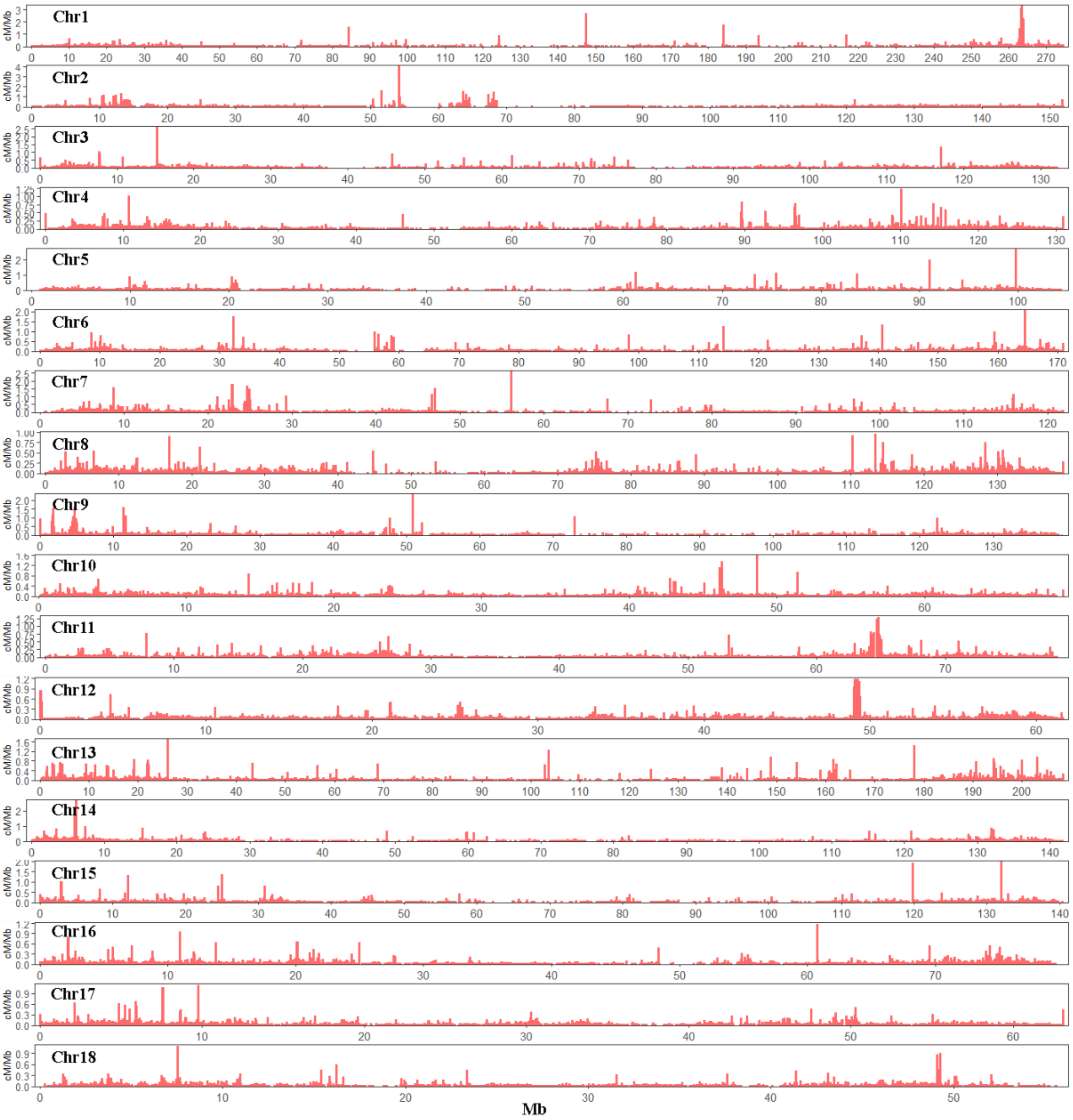

Figure S4 The fine recombination map of the SNP window was constructed using pyrho. The x-axis represents chromosomal positions, and the y-axis represents estimated recombination rates, with units in centimorgans per megabase (cM/Mb).

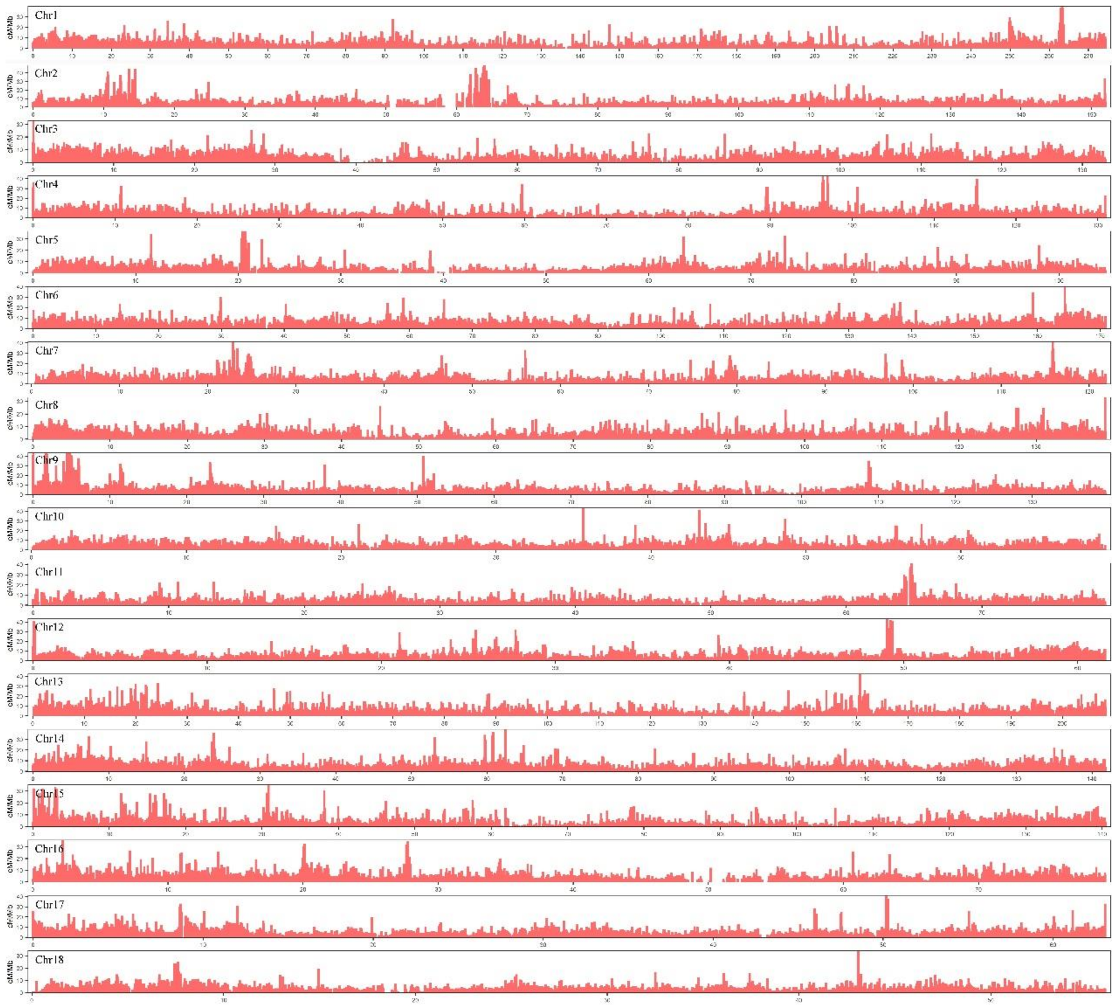

Figure S5 The fine recombination map of the SNP window was constructed using FastEPRR 5Kb window. The x-axis represents chromosomal positions, and the y-axis represents estimated recombination rates, with units in centimorgans per megabase (cM/Mb).

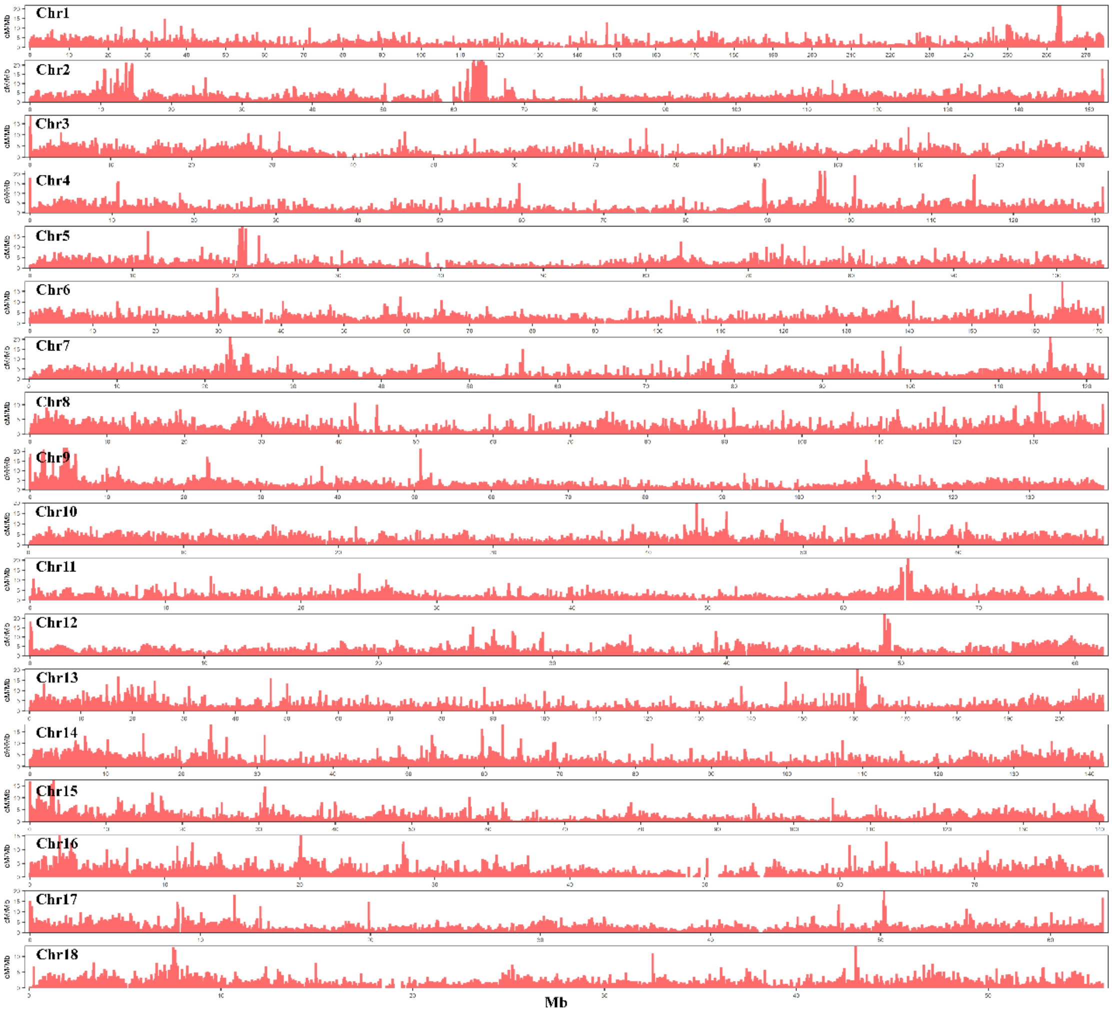

Figure S6 The fine recombination map of the SNP window was constructed using FastEPRR 10Kb window. The x-axis represents chromosomal positions, and the y-axis represents estimated recombination rates, with units in centimorgans per megabase (cM/Mb).

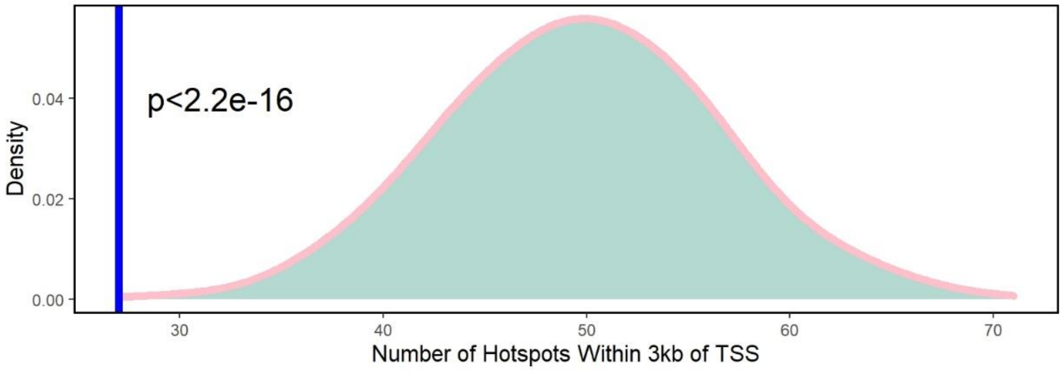

Figure S7 The number of recombination hotspots overlapping with the broad peaks of H3K4Me3 compared to 1000 simulated "randomly points". The pink line represents the distribution density of the overlap count between H3K4me3 broad peaks and 1000 randomly selected positions, while the blue line represents the number of recombination hotspots intersecting with H3K4me3 broad peaks. The P-value is calculated by T-test.
